# Surface roughness changes in bulk-fill resin composites after simulated toothbrushing with whitening toothpastes

**DOI:** 10.1186/s12903-026-08335-1

**Published:** 2026-04-16

**Authors:** Ceren Deger, Arzu Mujdeci̇

**Affiliations:** 1 Department of Restorative Dentistry, Faculty of Dentistry, Bezmialem Vakif University, Adnan Menderes Bulv. Fatih, Istanbul, Türkiye; 2 Department of Restorative Dentistry, Faculty of Dentistry, Ankara University, Ankara, Türkiye

**Keywords:** Whitening toothpastes, Bulk-fill resin composites, Surface roughness, Toothbrushing simulation, Relative dentin abrasivity (RDA)

## Abstract

**Background:**

Whitening toothpastes are widely used for the management of extrinsic tooth discolouration; however, their abrasive and chemical components may adversely affect the surface properties of resin based restorative materials. Surface roughness is a critical parameter influencing plaque accumulation, discolouration, and long-term aesthetic performance. Therefore, the aim of this in vitro study was to evaluate the effect of simulated toothbrushing with five different whitening toothpastes on the surface roughness of bulk-fill resin composites.

**Methods:**

Two bulk-fill resin composites (GrandioSO x-tra and Filtek Bulk Fill Posterior) and one nanohybrid composite (Clearfil Majesty Esthetic) were investigated (*n* = 60 per material). Specimens were randomly assigned to five whitening toothpaste groups (Opalescence Whitening, Colgate Optic White, Rocs Sensation Whitening, Signal White Now, and Curaprox Black Is White) and a distilled water control (*n* = 10). Simulated toothbrushing was performed using an electric toothbrush under a constant force of 2 N for 5 and 30 min, corresponding to approximately two months and one year of clinical brushing. Surface roughness (Ra, µm) was measured using a contact profilometer. Changes in roughness (ΔRa) were analyzed using two-way ANOVA and Bonferroni post-hoc tests (α = 0.05), with effect sizes calculated as partial eta squared (ηp²).

**Results:**

Toothpaste formulation, composite type, and their interaction significantly influenced surface roughness at both brushing durations (*p* < 0.001). After 5 min, significant differences were mainly observed for Clearfil Majesty Esthetic. After 30 min, surface roughness increased markedly for all composites, with very large interaction effects (ηp² = 0.832). Toothpastes containing optical agents, enzymatic components, or activated charcoal produced greater ΔRa values, whereas Opalescence Whitening showed changes comparable to the control in some groups. Mechanical brushing alone also resulted in measurable roughness increases.

**Conclusions:**

Whitening toothpastes significantly affect the surface roughness of bulk-fill resin composites in a material and time dependent manner. Prolonged brushing amplifies surface degradation, and dentin-based abrasivity indices alone may not fully explain or predict composite surface alterations. Clinicians should consider the potential long-term effects of whitening dentifrices on existing resin based restorations.

**Supplementary Information:**

The online version contains supplementary material available at 10.1186/s12903-026-08335-1.

## Background

In recent years, the increasing attention given to dental aesthetics has made tooth whitening one of the most frequently requested aesthetic procedures in dentistry [1]. This growing demand for whitening, together with the widespread availability of over-the-counter (OTC) whitening products, has led to a marked increase in products marketed directly to the public for the management of tooth discolouration. Among these OTC options, whitening toothpastes are particularly popular due to their ease of use, offering a practical approach to reducing enamel surface discolourations [2].

Specifically, whitening toothpastes are primarily intended to reduce or prevent extrinsic tooth discolouration (ETD), commonly referred to as staining, which negatively affects the tooth colour. ETD develops through the retention of colored substances within bacterial plaque and the acquired pellicle or through chemical modifications of these organic surface layers. The formation of ETD is influenced by factors such as chromogenic plaque bacteria, smoking habits, and frequent consumption of staining agents including coffee, tea, and red wine [3]. Inadequate toothbrushing and the use of dentifrices with insufficient abrasivity may facilitate the accumulation of stained pellicle and pigmented deposits; as such, a certain degree of abrasivity is considered necessary for the effective removal or prevention of extrinsic stains [4]. Relatedly, the whitening efficacy of toothpastes largely depends on the incorporated whitening agents, which may be classified as abrasive, chemical, or optical. Abrasives such as hydrated silica, perlite, and alumina predominantly contribute to the mechanical removal of surface stains, whereas enzymatic agents (e.g., papain and bromelain) support stain removal through protein degradation. Peroxide-containing formulations chemically modify chromogenic pigments adhered to the tooth surface, thereby reducing the discolouration intensity. Optical whitening dentifrices containing substances such as blue covarine or titanium oxide, change the perception of the tooth colour, creating the illusion of a whiter tooth [5]. Recently, activated charcoal has been added to dentifrices for the purpose of increasing the whitening efficacy and has gained popularity. However, scientific evidence supporting the whitening efficacy of activated charcoal products remains limited, and potential adverse effects have been reported, such as the marginal staining of resin-based restorations [6].

Resin composites are widely used for conservative restorations due to their ability to mimic the appearance of natural teeth. In particular, bulk-fill resin composites have gained popularity because they can be placed at increments of up to 4–5 mm, thus reducing the clinical time required for implementation in comparison with conventional incremental techniques, while also providing an adequate depth of cure and reduced polymerisation shrinkage stress [7, 8]. Nevertheless, the clinical longevity of these materials is closely related to their surface characteristics, including their colour stability, gloss, and surface roughness, which may be adversely affected by mastication, toothbrushing, and variations in oral temperature and pH [9].

In particular, toothbrushing with whitening toothpastes may adversely affect the surface of resin-based restorations due to the presence of abrasive particles and chemical agents capable of altering their surface properties [9–11]. These effects may increase the surface porosity of the resin and promote the loss of charged components, thus facilitating water absorption [12]. Consequently, reductions in the surface smoothness, gloss, and colour stability of the resin may occur, as surface roughness directly influences pigment accumulation [13]. Surface roughness is characterized by wear-induced grooves and scratches of varying shape, direction, and depth [14]. Therefore, evaluating the effects of whitening toothpastes on the surface roughness of restorative materials is of considerable clinical relevance.

Although previous studies have investigated the effects of whitening toothpastes on resin-based restorative materials, most have evaluated a limited number of formulations [14–17]. Therefore, the aim of this in vitro study was to evaluate the effects of multiple whitening toothpastes on the surface roughness of bulk-fill resin composites.

 The null hypotheses tested in this study were as follows:

1. different bulk-fill resin composite materials would not show significant differences in surface roughness after toothbrushing,
2. toothbrushing with different whitening toothpastes would not significantly affect the surface roughness of bulk-fill resin composites, and.
3. there would be no interaction effect of the composite type and toothpaste formulation on the surface roughness values.

## Methods

### Specimen size calculation

The sample size was calculated based on the estimated effect size between groups according to the previous literature [18]. Specifically, it was determined that at least *n* = 8 specimens were required in each subgroup for an estimated medium effect size of f = 0.30, with 80% power and a 5% type I error rate. Therefore, *n* = 10 specimens were included in each subgroup.

### Specimen preparation

The effect of simulated toothbrushing with five different whitening toothpastes on the surface roughness of two bulk-fill resin composites (GrandioSO x-tra, GSX and Filtek Bulk Fill Posterior, FBF) and one nanohybrid resin composite (Clearfil Majesty Esthetic, CME) was evaluated. The resin composite materials used in this study are listed in Table 1. For each composite material, 60 disc-shaped specimens (5 mm × 2 mm) were prepared using plastic molds. The specimens were covered with a Mylar strip and pressed with a glass slide to obtain a standardized smooth surface. After the removal of the glass slide, the specimens were light-cured for 20 s using an LED curing unit (Monitex Industrial Co. Ltd., Taipei, Taiwan) with an irradiance of 1200 mW/cm². All of the specimens were then stored in distilled water at 37 °C for 24 h prior to testing.

**Table 1:** Composition and characteristics of the resin composites used in the study

Resin Composite	Type	Resin matrix	Filler content
Clearfil Majesty Esthetic (CME) (Kuraray, Okayama, Japan)	Nanohybrid, incrementally filled	Bis-GMA, hydrophobic aromatic dimethacrylate	Silanated barium glass (mean particle size 0.7 μm) and pre-polymerized organic filler including nanofillers (78 wt%, 66 vol%)
Filtek Bulk Fill Posterior (FBF) (3 M ESPE, St. Paul, MN, USA)	Nanofilled bulk-fill	AUDMA, UDMA, 1,12-dodecane-DMA	Non-agglomerated silica (20 nm), zirconia (4–11 nm), aggregated zirconia/silica clusters, ytterbium trifluoride filler (76.5 wt%, 58.4 vol%)
GrandioSO x-tra Bulk Fill (GSX) (VOCO, Cuxhaven, Germany)	Nanohybrid bulk-fill	Bis-GMA, Bis-EMA, aliphatic dimethacrylate	Inorganic fillers (86 wt%, ~ 70 vol%*)

### Simulated toothbrushing procedure

Specimens from each composite group were randomly assigned to six subgroups (*n* = 10), corresponding to five whitening toothpastes (Opalescence Whitening, Colgate Optic White, Rocs Sensation Whitening, Signal White Now, and Curaprox Black Is White) and a distilled water control group. The whitening toothpastes used in this study are listed in Table 2. The simulated toothbrushing was performed using an electric toothbrush (Oral-B Pro Trizone 500, Procter & Gamble, Ohio, USA) mounted on a custom-made holder to ensure a constant brushing force of 2 N. The brushing was carried out using a standardized brush head (Oral-B CrossAction, Procter & Gamble, Ohio, USA) under slurry conditions, prepared at a toothpaste-to-distilled water ratio of 1:3 [18]. Specimens in the control group were brushed using distilled water only.

**Table 2:** Composition of the whitening toothpastes used in the study

Toothpaste	Ingredients
Colgate Optic White (Colgate-Palmolive, New York, NY, USA)	Sodium Monofluorophosphate, Glycerin, Calcium Pyrophosphate, Propylene Glycol, PEG/PPG-116/66 Copolymer, PEG-12, PVP, Tetrasodium Pyrophosphate, Sodium Lauryl Sulfate, Silica, Aroma, Sodium Saccharin, Phosphoric Acid, Hydrogen Peroxide, BHT, Limonene
Curaprox Black Is White (Curaden, Kriens, Switzerland)	Activated Carbon, Water, Sorbitol, Hydrated Silica, Glycerin, Carbon Black, Bentonite, Aroma, Decyl Glucoside, Sodium Monofluorophosphate, Cocamidopropyl Betaine, Tocopherol, Mica, Xanthan Gum, Nano-hydroxyapatite, Titanium Dioxide, Microcrystalline Cellulose, Maltodextrin, Acesulfame, Sodium Benzoate, Potassium Chloride, Potassium Sorbate, Menthyl Lactate, Methyl Diisopropyl Propionamide
Opalescence Whitening Toothpaste (Ultradent, South Jordan, UT, USA)	Glycerin, Water (Aqua), Silica, Sorbitol, Xylitol, Flavor (Aroma), Poloxamer, Sodium Lauryl Sulfate, Carbomer, FD&C Blue No.1 (CI 42090), FD&C Yellow No.5 (CI 19140), Sodium Benzoate, Sodium Hydroxide, Sparkle (CI 77019, CI 77891), Sucralose, Xanthan Gum
Rocs Sensation Whitening (Rocs, Munich, Germany)	Sorbitol, Silica, Glycerin, Water, Xylitol, Cocamidopropyl Betaine, Aroma, Xanthan Gum, Calcium Glycerophosphate, Bromelain, Magnesium Chloride, Sodium Saccharin, Sodium Benzoate, o-Cymen-5-ol, Titanium Dioxide
Signal White Now (Unilever, London, UK)	Sorbitol, Water, Hydrated Silica, Sodium Lauryl Sulfate, Cellulose Gum, Sodium Saccharin, Trisodium Phosphate, PVM/MA Copolymer, Sodium Fluoride, CI 74160

Ingredient lists were obtained from manufacturers’ product information

*PEG* polyethylene glycol, *PVP* polyvinylpyrrolidone, *PVM/MA* polyvinyl methyl ether/maleic acid

Each specimen was subjected to simulated toothbrushing for 5 min and 30 min. These durations were selected to represent approximately 2 months and 1 year of clinical toothbrushing, respectively, based on the assumption that the recommended daily toothbrushing time is approximately 120 s, corresponding to nearly 6 s of brushing per tooth surface per day. Similar simulation protocols have been reported in previous toothbrushing studies [19, 20]. To maintain consistent brushing efficacy, the toothbrush heads were replaced every 5 min throughout the procedure.

### Surface roughness measurement

Surface roughness measurements were performed using a contact profilometer (Mahr M2, Mahr GmbH, Göttingen, Germany) with a stylus force of 0.7 mN, a cut-off length (λc) of 0.25 mm, and a tracing length of 2 mm. Three measurements were obtained from different locations on each specimen surface, and the mean value of these measurements (Ra) was calculated and recorded as the baseline surface roughness. Following each brushing interval, the specimens were rinsed with distilled water and ultrasonically cleaned for 5 min to remove residual toothpaste. The surface roughness measurements were then repeated using the same protocol after both brushing periods (5 min and 30 min), and the resulting values were recorded as the post-brushing surface roughness.

### Statistical analysis

The data were analysed using IBM SPSS Statistics 22.0 software (IBM Corp., Armonk, NY, USA). The normality of the data distribution was assessed prior to analysis. Homogeneity of variances was assessed prior to implementing the two-way analysis of variance (ANOVA). Changes in the surface roughness values (ΔRa) were calculated as the differences between the baseline and post-brushing measurements. The effects of composite type, toothpaste formulation, and their interaction on surface roughness were analysed using an ANOVA, which was performed separately for the baseline to 5 min and baseline to 30 min brushing intervals.

When statistically significant differences were detected, Bonferroni post-hoc tests were applied for pairwise comparisons. The level of statistical significance was set at α = 0.05. The effect sizes were calculated using partial eta squared (ηp²) to determine the magnitude of the main and interaction effects.

## Results

The changes in surface roughness (ΔRa) values after simulated toothbrushing are presented in Tables 3 and 4. The ANOVA revealed that surface roughness was significantly influenced by toothpaste formulation, resin composite type, and the interaction between these factors at both brushing durations (*p* < 0.001).

**Table 3:** Changes in surface roughness (ΔRa, µm) after 5 min of simulated toothbrushing

	CME (mean ± SD)	GSX (mean ± SD)	FBF (mean ± SD)	Effect size (ηp²)	*p*
Distilled water	0.038 ± 0.009^a^	0.028 ± 0.006^a^	0.033 ± 0.013^a^	0.012	0.387
Opalescence Whitening Toothpaste	0.034 ± 0.010^a^	0.045 ± 0.013^ab^	0.034 ± 0.015^ab^	0.019	0.218
Rocs Sensation Whitening	0.077 ± 0.042^b^	0.031 ± 0.010^ab^	0.047 ± 0.009^ab^	0.204	< 0.001
Colgate Optic White	0.040 ± 0.012^a^	0.046 ± 0.016^b^	0.050 ± 0.014^ab^	0.012	0.382
Curaprox Black Is White	0.036 ± 0.018^a^	0.031 ± 0.009^ab^	0.046 ± 0.012^ab^	0.027	0.111
Signal White Now	0.078 ± 0.019^b^	0.031 ± 0.010^ab^	0.053 ± 0.019^b^	0.207	< 0.001
Effect size (ηp²)	0.342	0.070	0.076		
*p*	< 0.001	0.038	0.024		

Different superscript letters within the same row indicate statistically significant differences among composite materials (*p* < 0.05)

Effect sizes are expressed as partial eta squared (ηp²)

**Table 4:** Changes in surface roughness (ΔRa, µm) after 30 min of simulated toothbrushing

	CME (mean ± SD)	GSX (mean ± SD)	FBF (mean ± SD)	Effect size (ηp²)	*p*
Distilled water	0.106 ± 0.019^a^	0.058 ± 0.011^a^	0.070 ± 0.021^a^	0.130	< 0.001
Opalescence Whitening Toothpaste	0.081 ± 0.010^a^	0.074 ± 0.018^a^	0.085 ± 0.022^ab^	0.007	0.550
Rocs Sensation Whitening	0.272 ± 0.034^b^	0.068 ± 0.012^a^	0.155 ± 0.013^e^	0.715	< 0.001
Colgate Optic White	0.094 ± 0.013^a^	0.108 ± 0.030^b^	0.135 ± 0.022^cd^	0.094	< 0.001
Curaprox Black Is White	0.096 ± 0.020^a^	0.241 ± 0.032^c^	0.115 ± 0.025^bc^	0.598	< 0.001
Signal White Now	0.290 ± 0.034^b^	0.233 ± 0.028^c^	0.146 ± 0.024^de^	0.557	< 0.001
Effect size (ηp²)	0.849	0.810	0.411		
*p*	< 0.001	< 0.001	< 0.001		

Different superscript letters within the same row indicate statistically significant differences among composite material*s *(*p* < 0.05)

Effect sizes are expressed as partial eta squared (ηp²)

After 5 min of simulated toothbrushing, significant differences in surface roughness changes were observed depending on the toothpaste formulation and composite material (Table 3). A significant interaction effect between composite type and toothpaste formulation on changes in surface roughness was detected (*p* < 0.001).

When the effects of the different toothpastes were evaluated within each composite material, significant differences among the toothpaste formulations were mainly observed for Clearfil Majesty Esthetic, whereas GSX and FBF exhibited more limited differences across the toothpastes. Specifically, the effect of toothpaste formulation on surface roughness was most pronounced for CME, demonstrating a large effect size (ηp² = 0.342), whereas GSX (ηp² = 0.070) and FBF (ηp² = 0.076) exhibited moderate effect sizes.

Among the tested toothpastes, Rocs Sensation Whitening and Signal White Now resulted in significantly greater changes in surface roughness compared with the other toothpastes, particularly for Clearfil Majesty Esthetic (*p* < 0.001). No significant differences among the composite materials were detected for the distilled water, Opalescence Whitening, Colgate Optic White, or Curaprox Black Is White groups (*p* > 0.05).

After 30 min of simulated toothbrushing, the change in surface roughness increased markedly for all composites (Table 4). The ANOVA revealed significant main effects of toothpaste formulation and composite type, as well as a highly significant interaction effect between these factors (*p* < 0.001).

Toothpaste formulation exhibited a very large effect on changes in surface roughness for CME (ηp² = 0.849) and GSX (ηp² = 0.810) and a large effect for FBF (ηp² = 0.411). Clearfil Majesty Esthetic exhibited the highest surface roughness changes, especially when brushing with Rocs Sensation Whitening and Signal White Now (*p* < 0.001). For all of the resin composites, the surface roughness values obtained with Opalescence Whitening toothpaste were comparable to the distilled water control (*p* > 0.05). Notably, Opalescence Whitening was the only toothpaste that did not produce significant differences in surface roughness among the resin composites after 30 min of toothbrushing *(p* > 0.05).

Overall, the magnitude of surface roughness changes after 30 min of brushing varied considerably depending on both the toothpaste formulation and the composite material, reflecting a strong material-dependent response to whitening dentifrices.

## Discussion

This study evaluated the effects of different whitening toothpastes on the surface roughness of bulk-fill resin composites and demonstrated that both the toothpaste formulation and composite type significantly influenced the changes in surface roughness observed. Moreover, a strong interaction effect between these factors was observed, indicating that the effect of whitening toothpastes on surface roughness is highly material-dependent, particularly after prolonged simulated toothbrushing. Accordingly, the null hypotheses tested in the present study were rejected.

Surface roughness is a critical determinant of the clinical performance of composite restorations, as it is closely associated with both bacterial adhesion and discolouration risk [21]. The surface roughness is influenced by the intrinsic characteristics of the composite material, including the resin matrix composition and filler size and distribution, as well as by the finishing and polishing procedures used [22]. Although advances in micro and nano-filler technologies have improved the achievable surface smoothness of contemporary composites, the lowest roughness values obtained under ideal laboratory conditions are unlikely to be fully replicated in clinical practice [23]. Therefore, any additional surface alterations induced by external factors, such as toothbrushing with whitening dentifrices, may have important clinical implications relating to the longevity and aesthetic stability of composite restorations.

A surface roughness threshold of approximately 0.2 μm has been proposed, above which plaque accumulation and staining are more likely to occur, while lower surface roughness values are considered clinically acceptable [24]. In the present study, the surface roughness of the resins was evaluated using a contact profilometer, which is widely considered suitable for resin composite specimens due to its measurement accuracy, reproducibility, and practicality for quantitative analysis [25, 26].

Relative dentin abrasivity (RDA) is widely used as a standardized parameter to estimate the abrasive potential of dentifrices and is primarily based on dentin wear measurements according to ISO guidelines [27]. In this study, the RDA value of Signal White Now was not available, which limits direct comparisons among the studied toothpastes based on dentin abrasivity indices. As such, a direct correlation between a toothpaste’s RDA value and the observed surface roughness changes could not be carried out. Nevertheless, previous studies have demonstrated that the surface roughness of resin-based restorative materials is influenced not only by RDA values but also by the concentration, morphology, and physical characteristics of the abrasive particles in dentifrice formulations. Furthermore, abrasivity indices derived from dentin may not fully reflect the wear behavior of resin composites [28, 29].

In addition, Signal White Now contains blue covarine, an optical whitening agent designed to deposit a surface coating that modifies light reflection [15]. Such pigments have been reported to form heterogeneous surface films, which may preferentially accumulate within the micro-irregularities of composite surfaces and be detected as increased roughness in profilometric measurements. Furthermore, depending on the microstructural characteristics of the composite material (e.g., the resin matrix composition and filler distribution), early matrix wear and filler particle detachment may further exacerbate changes in surface roughness following toothbrushing with whitening dentifrices [16].

Similarly, the greater changes in surface roughness observed for ROCS Sensation Whitening compared to other toothpastes further support the notion that these changes in resin-based restorative materials cannot be attributed solely to dentin abrasivity indices. ROCS Sensation Whitening contains hydrated silica as an abrasive component in combination with bromelain, a proteolytic enzyme that contributes to stain removal by degrading the organic components of the acquired pellicle [30]. Although bromelain is not classified as a conventional abrasive agent, its enzymatic activity may indirectly facilitate surface degradation by weakening the organic matrix at the composite surface [31], thereby increasing its susceptibility to mechanical wear during toothbrushing. This mechanism may also explain the significant surface roughness changes observed for Clearfil Majesty Esthetic after short-term brushing, as nanohybrid resin composites with a higher resin matrix content may be more vulnerable to early matrix degradation when exposed to enzymatic agents [30].

After the more prolonged simulated toothbrushing, the surface roughness increased markedly for all of the resin composites, indicating that brushing duration plays a critical role in cumulative surface deterioration. Bulk-fill resin composites are formulated with modified resin matrices and filler systems to provide an increased depth of cure, but these characteristics may render them susceptible to long-term abrasive challenges [32]. As brushing time increased, the combined effects of enzymatic matrix degradation, abrasive particle interaction, and filler–matrix debonding likely became more pronounced, resulting in greater changes in surface roughness. These findings reinforce previous studies indicating that the effects of whitening dentifrices on restorative materials are highly material- and time-dependent [30, 33].

In the present study, Curaprox Black Is White toothpaste resulted in one of the highest changes in surface roughness after 30 min of simulated toothbrushing, particularly for GrandioSO x-tra, showing a pattern comparable to that observed for Signal White Now. This finding may be attributed to the presence of activated charcoal particles in the formulation, which differ substantially from conventional abrasives such as hydrated silica in terms of their particle morphology, size distribution, and surface characteristics. Indeed, activated charcoal particles are typically irregular, angular, and heterogeneous, with a high specific surface area, and have been reported to induce micro-cutting and ploughing effects on restorative material surfaces rather than uniform polishing during prolonged brushing [11, 34].

Previous studies have demonstrated that charcoal-containing dentifrices can cause pronounced increases in the surface roughness of resin-based restorative materials, particularly under extended brushing protocols, even when the dentin abrasivity values are within moderate limits [11, 32]. This pattern suggests that dentin-based abrasivity indices may underestimate the abrasive potential of activated charcoal on composite surfaces, potentially because charcoal particles interact differently with resin matrices and inorganic fillers compared with dentin substrates. The significant susceptibility of GrandioSO x-tra observed in the present study may be related to its bulk-fill formulation and nanohybrid filler system with a high filler load, since preferential resin matrix wear around the densely packed fillers may lead to filler protrusion and subsequent filler–matrix debonding. Under extended toothbrushing, the repeated interaction between irregular charcoal particles and the exposed filler–matrix interface may then further exacerbate these wear processes, resulting in greater changes in surface roughness. Collectively, these findings highlight that the effects of whitening dentifrices on composite surface roughness are both material- and time-dependent [11, 32, 34, 35].

Notably, measurable increases in surface roughness of the resins were observed even in the distilled water control group after prolonged brushing. This finding highlights that the action of mechanical brushing alone, independent of dentifrice abrasivity or chemical composition, can induce surface degradation over time. Such results underscore the cumulative role of brushing duration and mechanical stress in composite surface deterioration and should be considered when interpreting the effects of whitening dentifrices [18].

This study has certain limitations that should be acknowledged. As an in vitro investigation, the experimental conditions cannot fully reproduce the complex oral environment, including the influence of saliva, pellicle formation, thermal variations, and individual brushing habits. Surface roughness was evaluated using contact profilometry, which enables reliable quantitative comparisons; however, additional techniques such as scanning electron microscopy (SEM) or three-dimensional surface analyses could provide further insight into surface morphology. Nevertheless, the primary aim of this study was to perform a standardized comparative analysis rather than detailed morphological characterization. Furthermore, toothbrushing was performed using toothpaste diluted with distilled water [36], which may not fully reflect clinical conditions. The presence of saliva, including ions and enzymes, may influence abrasive interactions and potentially reduce the effect of abrasives on restorative materials. In addition, RDA values were not available for all tested toothpastes, limiting direct comparisons based on abrasivity indices. Finally, the brushing protocol simulated short- and medium-term use; therefore, further studies incorporating longer-term simulations and advanced surface characterization methods are needed to better understand the clinical implications of these findings.

## Conclusions

- Whitening toothpaste formulation and resin composite type significantly influenced surface roughness changes after simulated toothbrushing.
- A strong interaction between toothpaste type and composite material was observed, indicating that the surface response to whitening dentifrices is material-dependent.
- Toothpastes containing optical agents, enzymatic components, or charcoal particles generally produced greater surface roughness changes.
- Prolonged brushing duration intensified surface roughness changes, including those induced by mechanical brushing alone.

Clinically, the long-term use of whitening toothpastes may adversely affect the surface integrity of resin-based restorations, and this should be considered when recommending whitening dentifrices to patients with existing composite restorations.

## Supplementary Information

Supplementary Material 1.

## Data Availability

All data generated or analysed during this study are included in this published article.

## Abbreviations

ANOVA: Analysis of variance
CME: Clearfil Majesty Esthetic
ΔRa: Change in surface roughness
ETD: Extrinsic tooth discolouration
FBF: Filtek Bulk Fill Posterior
GSX: GrandioSO x-tra
ISO: International Organization for Standardization
LED: Light-emitting diode
N: Newton
OTC: Over-the-counter
Ra: Arithmetic mean surface roughness
RDA: Relative dentin abrasivity
SEM: Scanning electron microscopy
ηp²: Partial eta squared
