# Supplementary material for: Surface roughness changes in bulk-fill resin composites after simulated toothbrushing with whitening toothpastes
Source: BMC Oral Health. 2026 Apr 16;26:981. doi: 10.1186/s12903-026-08335-1 (PMC13248276; doi:10.1186/s12903-026-08335-1)
Supplement: Supplementary file 1 — Supplementary Material 1. [file 12903_2026_8335_MOESM1_ESM.pdf]

Scribbr

Declaration of Professional Editorial Assistance

Scribbr hereby declares to have undertaken professional editorial work in the preparation of the document of **Ceren Deger** according to the Scribbr Improvement Model.

Proofread by

The document was edited by **Amelia**.

Scribbr Guarantees

Scribbr editors are all native speakers who must comply with strict editing guidelines.

1. The professional editorial intervention is restricted to:
    - Language
    - Consistency
    - Academic style
2. When Scribbr editors provide advice on structure or clarity, they do so by providing examples only and not by undertaking a structural re-write themselves.
3. The mark-up is done using Track Changes. No clean document is provided, so the student always needs to process all changes manually.
4. If an editor suspects plagiarism, the document will not be proofread.

Acknowledged by

Name of Author: **Ceren Deger**

Work Title: **Surface Roughness Changes in Bulk-Fill Resin Composites After Simulated Toothbrushing with Whitening Toothpastes**

Scribbr CEO Bas Swaen: I declare that this academic paper has been proofread in compliance with the abovementioned conditions.

Date: March 21, 2026

Signature: Bas Swaen
